# Bacteriophage-Derived Peptidase CHAP_K_ Eliminates and Prevents Staphylococcal Biofilms

**DOI:** 10.1155/2013/625341

**Published:** 2013-02-03

**Authors:** Mark Fenton, Ruth Keary, Olivia McAuliffe, R. Paul Ross, Jim O'Mahony, Aidan Coffey

**Affiliations:** ^1^Department of Biological Sciences, Cork Institute of Technology, Bishopstown, Cork, Ireland; ^2^Teagasc, Moorepark Food Research Centre, Fermoy, County Cork, Ireland

## Abstract

New antibacterial agents are urgently needed for the elimination of biofilm-forming bacteria that are highly resistant to traditional antimicrobial agents. Proliferation of such bacteria can lead to significant economic losses in the agri-food sector. This study demonstrates the potential of the bacteriophage-derived peptidase, CHAP_K_, as a biocidal agent for the rapid disruption of biofilm-forming staphylococci, commonly associated with bovine mastitis. Purified CHAP_K_ applied to biofilms of *Staphylococcus aureus* DPC5246 completely eliminated the staphylococcal biofilms within 4 h. In addition, CHAP_K_ was able to prevent biofilm formation by this strain. The CHAP_K_ lysin also reduced *S. aureus* in a skin decolonization model. Our data demonstrates the potential of CHAP_K_ as a biocidal agent for prevention and treatment of biofilm-associated staphylococcal infections or as a decontaminating agent in the food and healthcare sectors.

## 1. Introduction

Staphylococcal species commonly colonise the skin and mucosal membranes of both humans and animals. They are a significant causative agent of bovine mastitis in dairy herds [1] and are also associated with a number of diseases in humans, ranging from a variety of skin conditions to more serious infections such as septicemia [2]. Staphylococcal food poisoning is among the most common food-borne microbial diseases [3] and contamination of food industrial surfaces with staphylococcal species has been demonstrated to be a considerable risk factor [4–6]. Along with the urgent requirement for novel antibacterials to combat the prevalence of antibiotic/disinfectant resistant staphylococci in food processing, veterinary and healthcare settings, there is an increasing need for effective antimicrobial agents which can prevent and treat staphylococcal biofilm-associated infections [7–11]. 

Biofilms are multilayered communities of sessile cells protected by an extracellular matrix, which often adhere to food contact surfaces, damaged tissue and indwelling medical devices [12–14]. Once formed, biofilms may be up to 1,000 times more resistant to antimicrobial agents than planktonic cells alone making them particularly difficult to eliminate [15]. This can ultimately lead to increased risk of persistent infections, as is commonly the case with bovine mastitis [16]. In addition, because of their increased levels of resistance, biofilm-associated infections can result in a need for explantation of medical devices in human healthcare settings [17, 18]. Although the precise mechanisms of biofilm antibiotic resistance have yet to be fully resolved, failure to successfully treat infections with conventional therapies necessitates the investigation and development of novel treatment strategies [9, 18, 19].

In recent years, bacteriophage endolysins (phage lysins) have been the focus of research into combatting antibiotic resistance in Gram-positive pathogens [20–24]. These cell wall peptidoglycan hydrolases possess a number of advantages over conventional antibiotics including, rapid lytic activity against bacterial cells, low probability of developing bacterial resistance, and significantly lower chance of disrupting commensal microflora, due to the enzymes' specificity [25–28]. 

Our group previously reported the ability of phage K and modified derivatives to prevent biofilm formation and to reduce established biofilm density [29]. However, endolysins have a number of advantages over using whole phage as antimicrobial agents. In the case of whole phage, resistance arising from either adsorption inhibition, restriction modification and abortive infection have been reported in many genera [30–32]. Bacteriophage and their hosts have coevolved over millions of years. The equilibrium in this relationship has been maintained by continual development of resistance and counter resistance. In contrast, with the use of phage endolysins, there has been no report of bacteria developing resistance to these lytic agents even after extensive growth of the bacterium in the presence of sublethal levels of enzyme [25, 33, 34]. In addition, by using phage endolysins instead of whole phage the risk of horizontal gene transfer of virulence genes is avoided. Phage preparations also have the possibility of containing exo- and endotoxins from their respective bacterial host. By overexpressing an endolysin in well-characterised avirulent laboratory strains this risk is circumvented. 

To date a number of phage lysins have been described which demonstrate activity against staphylococci including LysK [35], LysWMY [36], Φ11 lysin [37], MV-L [38], LysH5 [39], LysGH15 [40], PlyV12 [41], SAL-2 [42], and SAL-1 [43]. Producing tailor-made proteins by combining domains from phage lysins with various other domains has also been investigated[28, 44, 45]. Multidomain lysins have previously been examined for control of biofilms namely Φ11 lysin [37] and SAL-2 [42]. This is the first report of a single domain lysin being used for this purpose. Cysteine, histidine dependant amidohydrolase/peptidase CHAP_ K_ (18.6 kDa) is a truncated derivative of a phage lysin (LysK, 54 kDa) from the myoviridae staphylococcal phage K [35, 46]. This endopeptidase specifically cleaves the peptide bond between D-alanine and the first glycine in the pentaglycine cross-bridge of staphylococcal cell wall peptidoglycan [47]. We have previously reported that purified CHAP_ K_ exhibits stronger activity than the original multidomain lysin (LysK) [46] and displays rapid lytic activity against a variety of pathogenic staphylococcal species including *Staphylococcus epidermidis *and methicillin-resistant *S. aureus* (MRSA) strains [46, 48]. Here we examine the efficacy of the phage-derived CHAP_ K_ enzyme in eliminating *Staphylococcus aureus* biofilms and demonstrate its efficacy for removal of *S. aureus* from skin.

## 2. Materials and Methods

### 2.1. Bacterial Strains

The strains used in this study were *S. aureus* strain DPC5246 (Moorepark Food Research Centre, Teagasc, Fermoy, Cork, Ireland) and *S. aureus* strain Xen29 (Caliper Lifesciences, UK). Strain DPC5246 is an *S. aureus* bovine mastitis isolate [49]. Strain Xen29 is a confirmed biofilm producing pleural fluid isolate derived from the parental strain *S. aureus* ATCC 12600 [50]. It has been engineered to emit luminescence when metabolically active [51, 52]. Both strains were stored at −80°C and routinely grown on tryptic soya agar (TSA), in tryptic soya broth (TSB) or TSB supplemented with 1% D-(+)-glucose (TSBg) at 37°C. All media was supplied by Sigma-Aldrich. 

### 2.2. Production of CHAP_K_

CHAP_ K_ is comprised solely of the lytic CHAP domain of the anti-staphylococcal bacteriophage endolysin, LysK [35]. In a previous study by our group, the truncated phage lysin gene was cloned untagged into a pQE60 expression vector (Qiagen) and overexpressed in *Escherichia coli* (*E. coli*) XL1-Blue [46]. Highly active CHAP_ K_ (18.6 kDa) was purified to >90% homogeneity by cation exchange chromatography. The protein was then desalted and concentrated using an amicon ultra centrifugal filter (Milipore) with 10 kDa cut-off and subsequently stored at −80°C in 25 mM Tris pH 7. The lytic activity of CHAP_ K_ against live planktonic cells of staphylococci including multi-antibiotic resistant strains of clinical origin has been demonstrated previously [46, 53].

### 2.3. Staphylococcal Biofilm Reduction Using CHAP_K_

#### 2.3.1. Plate Staining Assay

A modified static microtitre plate assay, based on previous studies [54], was used to analyse biofilm formation and treatment with CHAP_ K _. Briefly, overnight (18–24 hr) colonies of *S. aureus* DPC5246 from a TSA plate were suspended in sterile ringers to an optical density equivalent to 0.5 McFarland standard and subsequently diluted 1 : 100 in TSBg to give a starting inoculum of 1.29 × 10^6^ CFU mL^−1^. In the biofilm disruption assay, 200 *μ*L volumes of the prepared culture were aliquoted into wells of a sterile 96-well microtitre plate (Sarstedt) and incubated at 37°C for 24 h. After this incubation period, wells were washed three times with 200 *μ*L of sterile ringers using a multichannel pipette (Gilson) to remove media and planktonic cells. Biofilm containing wells were then treated with 200 *μ*L of various concentrations of CHAP_ K_ (3.91–500 *μ*g mL^−1^) in sterile 25 mM Tris pH 7 or with 200 *μ*L of sterile 25 mM Tris pH 7 alone (control), at 37°C for 4 h. At the end of treatment all wells were washed again before the plate was inverted and left to dry for 1 h at 60°C. The biofilms were then stained with 200 *μ*L of 0.5% crystal violet solution for 15 min. The stain solution was removed and the wells were gently washed as before. The plate was left to dry, after which, 30% acetic acid were added to solubilise the stain. The biofilm disrupting ability of CHAP_ K_ was determined by examining the optical density of the wells spectrophotometrically. 

#### 2.3.2. Viability Plate Count Assay

A 96-well microtitre plate/peg-lid assay, based on the method used by Moskowitz et al. [55] was used to investigate if CHAP_ K_ can completely eliminate a staphylococcal biofilm. A peg-lid plate was used in order to ensure that the maximum number of cells were removed from experimental wells with the same efficiency. This method permits removal of the biofilm matrix by centrifugation prior to plating. Briefly, an overnight colony (18–24 h) of *S. aureus* DPC5246 was suspended in sterile Ringers to an optical density equivalent to 0.5 McFarland standard and subsequently diluted 1 : 100 in TSBg to give a starting inoculum of 1.29 × 10^6^ CFU/mL. 200 *μ*L of the TSBg cell suspension was transferred to the wells of a 96-well plate. As a negative control for biofilm formation, 200 *μ*L of TSBg was used. A peg lid was added and the plate was incubated statically for 24 h at 37°C [56]. After incubation the peg lid was removed and washed three times by placing it in a 96-well plate containing sterile ringers for 30 sec each time. 200 *μ*L of CHAP_ K_ at concentrations ranging from 125–1000 *μ*g mL^−1^ (diluted in 25 mM Tris pH7) was added to treatment wells. 200 *μ*L 25 mM Tris pH 7 was added to the control wells. The biofilm peg lid was placed on the antimicrobial challenge plate and incubated for 4 h at 37°C. After incubation the peg lid was washed three times in sterile ringers as before. Finally the lid was placed in a plate containing 200 *μ*L sterile ringers in each well and centrifuged at 800 g for 20 mins to remove any biofilm remaining on the pegs. Serial dilutions were performed on the contents of each well and a viable plate count was performed using Baird Parker Agar supplemented with egg yolk tellurite. 

### 2.4. Biofilm Prevention by CHAP_K_

#### 2.4.1. Plate Staining Assay

In order to investigate the ability of CHAP_ K_ to prevent the formation of *S. aureus *biofilms on artificial surfaces, the staining assay, as described previously for the biofilm reduction assay, was carried out with the following modification. At the beginning of the assay 100 *μ*L of CHAP_ K _, at concentrations ranging from 0.78 to 125.0 *μ*g mL^−1^, were added to 100 *μ*L of TSBg with 1.3 × 10^6^ CFU mL^−1^ of DPC5246 cells, in a sterile 96-well microtitre plate and incubated for 24 h at 37°C. 

#### 2.4.2. Viable Plate Count Assay

The ability of CHAP_ K_ to prevent biofilm formation was also investigated using a method similar to the viable plate count method described previously with the following changes. At the beginning of the assay 100 *μ*L of CHAP_ K _, at concentrations ranging from 0.78 to 125.0 *μ*g mL^−1^, were added to 100 *μ*L of TSBg with 1.3 × 10^6^ CFU mL^−1^ of DPC5246 cells, in a sterile 96-well microtitre plate.

### 2.5. Skin Decolonization Assay

This study was carried out using a modified version of the spray test of Hoopes et al. [57]. Briefly, three individual areas, 25 cm^2^ in size, were marked out on a section of porcine skin (obtained fresh from an abattoir). Each area was disinfected with 70% isopropyl alcohol wipes and allowed to dry at room temperature for up to 30 min. All three marked areas were then seeded with 100 *μ*L of 6.2 × 10^7^ CFU mL^−1^ (2.5 × 10^5^ CFU cm^−2^) of *S. aureus* DPC5246 by pipette, distributed evenly within each area with a sterile plastic spreader (Sarstedt) and allowed to dry for 30 min. CHAP_ K_ (200 *μ*g mL^−1^ in sterile H_2_O) was then misted 20 cm above one of the 25 cm^2^ areas, in two passes. The two remaining sections served as controls where one 25 cm^2^ area was misted with sterile H_2_O and the other was left untreated. The skin was then left to dry at room temperature for 30 min. Sterile cotton tipped swabs (Deltalab sterile swabs, Fisher Scientific, Ireland) were moistened in sterile Ringer's solution and used to sample each section of skin by rotating and rubbing the swab, in a zigzag pattern, and repeating at right angles. The tips of each swab were placed in 10 mls of Ringers solution and vigorously mixed using a vortex mixer to dislodge cells. The suspensions were serially diluted and plated on Baird Parker agar supplemented with egg yolk tellurite for enumeration of surviving cells. The work was also similarly done using the bioluminescent producing *S. aureus* Xen29 strain.

## 3. Results

### 3.1. Staphylococcal Biofilm Reduction Using CHAP_K_

#### 3.1.1. Plate Staining Assay

**Figure 1:**
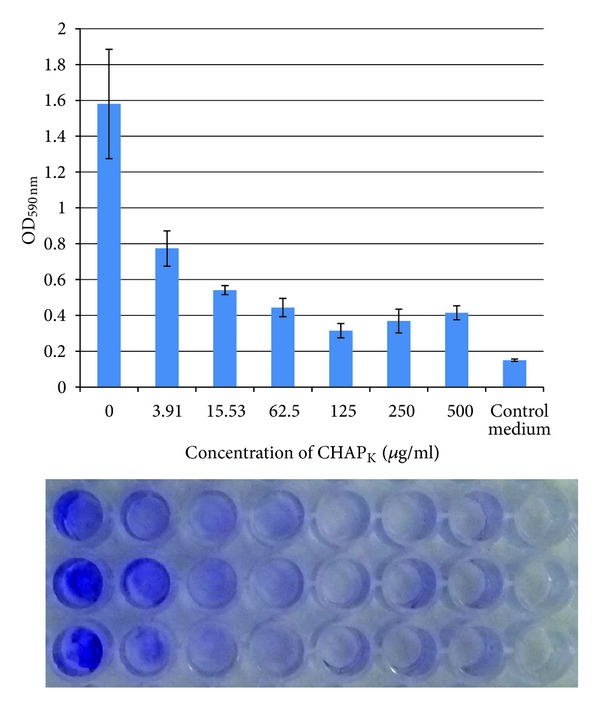
Biofilm staining assay demonstrating disruption of 24 h biofilms of *S. aureus* DPC5246 after 4 h incubation with CHAP_ K_ at concentrations ranging from 3.91–500 *μ*g/mL. The assay was carried out in triplicate in a flat bottomed 96-well plate. Absorbance readings at 590 nm (OD_590 nm_) are represented in the graph as the mean ± SE. Below the graph is a picture of the stain in the wells prior to solubilisation with acetic acid. The wells correspond to the data represented directly above each set.

A strong biofilm of *S. aureus* DPC 5246 was routinely formed when the strain was grown in TSB supplemented with 1% D-(+)-glucose for 24 hr at 37°C. This is represented by the strong staining seen in the untreated well in Figure 1. Solubilising of crystal violet stain and subsequent measurement of OD_590 nm_ allowed accurate quantification of staining and comparison between control and enzyme-treated wells. The data shown in the bar chart in Figure 1 represents the OD_590 nm_ of triplicate wells ± standard error. Mature biofilms (24 h) were treated with enzyme at concentrations ranging from to 3.91–500 *μ*g mL^−1^, for 4 h at 37°C. The OD_590 nm_ data for the biofilm disruption staining assay demonstrated that at all concentrations tested, CHAP_ K_ treatment reduced biofilm formation relative to the untreated control well (Figure 1). A one-way ANOVA indicated that CHAP_ K_ treatment caused a statistically significant change in biofilm formation (*P* value < 0.001). It is clear from the graph in Figure 1 that CHAP_ K_ successfully disrupted the *S. aureus *biofilms in a concentration dependant manner. Visual inspection of the degree and intensity of staining in the CHAP_ K_ treated wells compared to untreated biofilm wells indicated that even at a concentration as low as 3.91 *μ*g mL^−1^, CHAP_ K_ caused a visible reduction in biofilm mass. At a concentration of 62.5 *μ*g mL^−1^  CHAP_ K_ there was little or no visibly detectable staining of the wells (Figure 1).

#### 3.1.2. Viable Plate Count Assay

**Figure 2:**
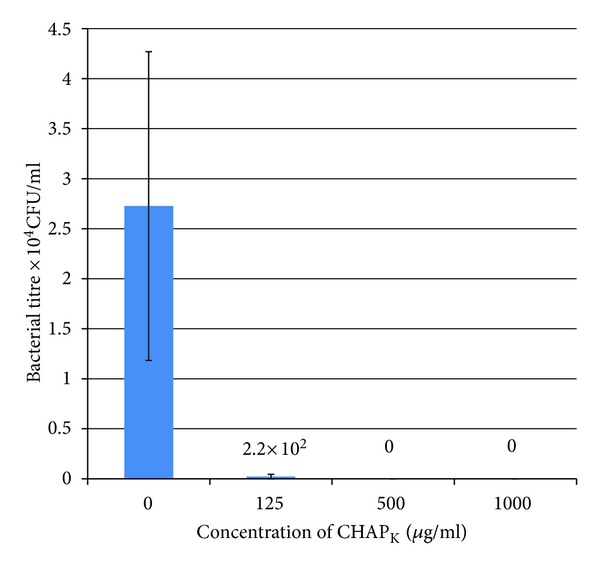
Viable plate count assay demonstrating the antimicrobial effect of a CHAP_ K_ treatment on 24 h biofilms of *S. aureus* DPC5246. 24 h staphylococcal biofilms were grown on peg lids in microtitre plates. The bar chart shows the CFU mL^−1^ that were retrieved from the pegs after 4 h treatment with CHAP_ K _, at the concentrations outlined below each bar. The assay was performed in triplicate and each bar is a representation of the mean ± SE. The values indicated above the bars are the mean CFU/mL after treatment.

Purified CHAP_ K_ ranging in concentration from 125–1000 *μ*g/mL, was used to treat a 24 h staphylococcal biofilm. The results of the viable plate counts are summarised in the bar chart in Figure 2, where each bar represents the average of triplicate plate counts ± standard error. After treatment with 125 *μ*g mL^−1^ a 2-log decrease was seen in the number of cells in the biofilm matrix on the pegs. The average plate count from the wells with untreated DPC5246 biofilms was 2.7 × 10^4^ CFU mL^−1^. The average viable plate count for the wells treated with 125 *μ*g mL^−1^ was 2.2 × 10^2^ CFU mL^−1^. After treatment with CHAP_ K_ at concentrations of 500 *μ*g mL^−1^ or higher there was complete eradication of the biofilm which corresponded to a 4-log drop in CFU mL^−1^ when compared to the untreated control wells. 

### 3.2. Biofilm Prevention by CHAP_K_

#### 3.2.1. Plate Staining Assay

**Figure 3:**
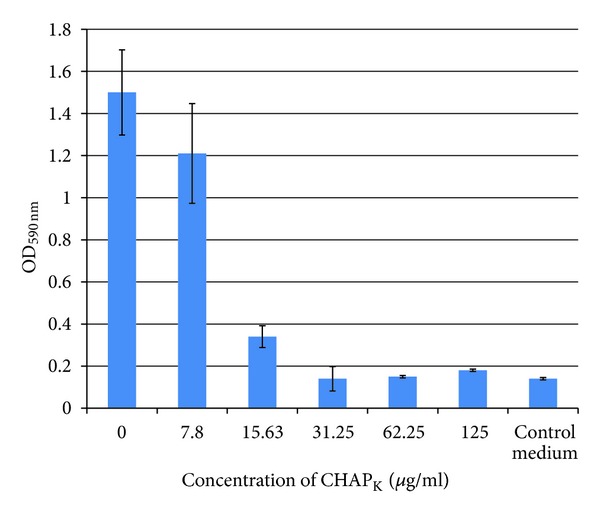
CHAP_ K_ biofilm prevention assay: staining. Concentrations of enzyme ranging from 0–125 *μ*g mL^−1^ were incubated with *S. aureus* DPC5246 in TSBg at 37°C for 24 h. The wells were stained with crystal violet (1%) and subsequently the stain was solubilised with acetic acid. Optical density readings at 590 nm (OD_590_) of all wells were recorded in a microtitre plate reader and displayed on the bar chart. Assays were carried out in triplicate and OD_590_ data was expressed as the mean ± SE.

To investigate the capacity of CHAP_ K_ to inhibit the formation of *S. aureus *biofilms, various concentrations of the enzyme were incubated with strain DPC5246 for 24 h at 37°C in a microtitre plate assay. After staining and subsequent solubilisation of stain, OD_590 nm_ measurements were recorded and used to assess the ability of CHAP_ K_ to prevent biofilm formation. This data is presented in Figure 3. A one-way ANOVA indicated that CHAP_ K_ treatment caused a statistically significant change in biofilm formation (*P* value < 0.001). Increasing degrees of biofilm prevention were evident in the presence of increasing concentrations of enzyme. At 15.63 *μ*g mL^−1^ a considerable decrease in optical density is seen when compared with the untreated control wells. A concentration of 31.25 *μ*g mL^−1^ indicated complete prevention as the mean OD_590 nm_ value (0.14) is exactly the same as that of the control medium.

#### 3.2.2. Viable Plate Count Assay

**Figure 4:**
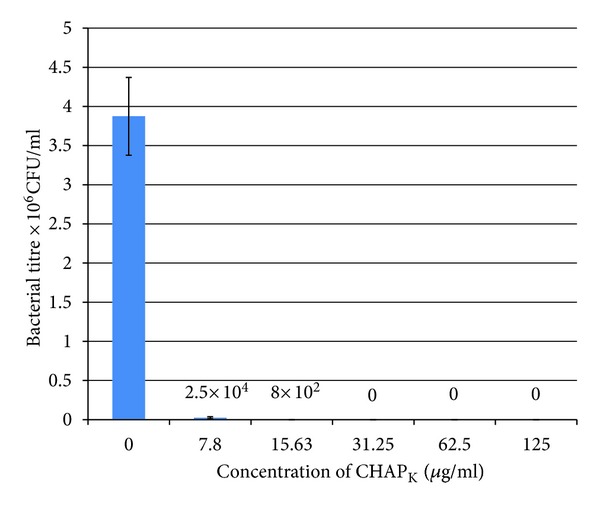
CHAP_ K_ biofilm prevention assay: viable plate count. Concentrations of enzyme ranging from 7.8−125 *μ*g mL^−1^ were incubated with *S. aureus* DPC5246 in TSBg at 37°C for 24 h in a 96 well peg plate. The pegs were washed after incubation and subsequently any cells remaining adhered to the pegs were removed by centrifugation at 800 rpm for 20 mins. Assays were carried out in triplicate and OD_590_ data was expressed as the mean ± SE. The values indicated above the bars are the mean CFU/mL after treatment.

To confirm that CHAP_ K_ is able to completely prevent biofilm formation a plate count assay was performed on wells in which *S. aureus* was grown in TSBg at various concentrations of CHAP_ K _. The bar chart in Figure 4 represents averages of triplicate values ± standard error. Growth of bacteria in the presence of 7.8 *μ*g mL^−1^  CHAP_ K_ caused a 2-log drop in biofilm formation and at 15.63 *μ*g mL^−1^ a 4-log reduction was evident. Complete prevention corresponding to a 6-log drop was achieved when the DPC 5246 was incubated with CHAP_ K_ at a concentration of 31.25 *μ*g mL^−1^ or higher.

### 3.3. Removal of *S. aureus* from Skin Using CHAP_K_

**Figure 5:**
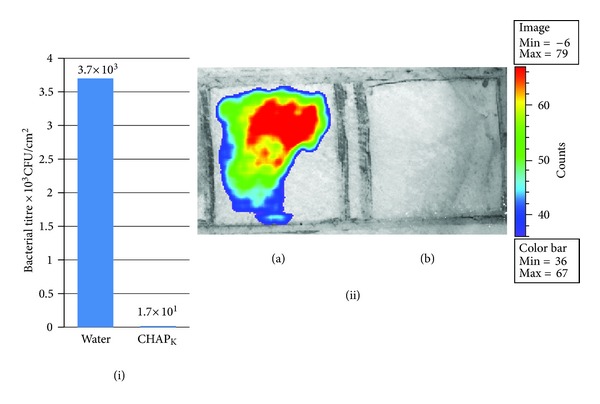
Skin decolonisation. Reduction of *S. aureus* on porcine skin using CHAP_ K _. (i) Two sections of porcine skin were seeded with 2.5 × 10^5^ CFU cm^−2^ of *S. aureus* DPC 5246. Plate counts were performed to enumerate the *S. aureus* present on porcine skin 30 min after being sprayed with either sterile water or CHAP_ K_ solution. (ii) Two sections of skin were seeded with 2.4 × 10^6^ CFU cm^−2^ of *S. aureus* Xen29. One area of skin was treated with sterile water (a) and the other area treated with CHAP_ K_ solution (b). Skin was imaged (exposure: 15 seconds, binning: medium, colour-scale 36–67) 30 min after treatment with CHAP_ K _/H_2_O using the *in vivo* Imaging System (IVIS) (Xenogen, Ca). This system enabled direct monitoring of the effect of CHAP_ K_ treatment in real time. The colour bar represents bacterial bioluminescence signal intensity, with red to blue indicating high and low bioluminescence, respectively.

The potential of CHAP_ K_ as a skin decolonization agent was assessed by incorporation of the enzyme into a spray. Sections of porcine skin (25 cm^2^) were seeded with 2.5 × 10^5^ CFU cm^−2^ of *S. aureus* strain DPC5246 and misted with 200 *μ*g mL^−1^ solution of CHAP_ K_ for two seconds. Water-treated and untreated skin sections had similar CFU values when enumerated after 30 minutes. This contrasted with a significant reduction in CFUs on the CHAP_ K _-treated skin, which received approximately 60 *μ*g of enzyme. CHAP_<?brm?>K<?erm?>_ treatment was found to be sufficient to remove >99% of *S. aureus* DPC5246 in 30 min when compared with treatment with water, i.e., reduced from 3.7 × 10^3^ CFU mL^−1^ when treated with water to 1.7 × 10^1^ CFU mL^−1^ when treated with the CHAPK solution. Similar results were achieved when CHAP_ K_ was employed against the bioluminescent strain *S. aureus* Xen29 (Figure 5). 

## 4. Discussion

Biofilms are recognised as a significant problem in the food industry. Biofilm-forming bacterial strains are generally much more difficult to kill than their planktonic counterparts. They survive in sub-optimal environmental conditions, display widespread resistance to antibiotics and disinfectants and often lead to persistent infections such as is commonly seen with bovine mastitis [16]. Biofilms may also interfere with various processes in food technology and engineering. For example, biofilms can impede liquid flow and heat transfer and lead to increased corrosion rates which can lead to economic losses [58]. 

This study demonstrates that the phage-derived peptidase, CHAP_ K _, can completely remove a mature staphylococcal biofilm in under 4 h and can also prevent establishment of a staphylococcal biofilm. In the biofilm context it is likely that CHAP_ K_ rapidly lyses sessile staphylococcal cells with an efficiency that brings about destabilization of the biofilm matrix leading to their subsequent detachment from solid surfaces. For formation of a mature staphylococcal biofilms a 24 h incubation period is commonly used [37, 56, 59, 60]. In the results presented in this study the untreated wells in the biofilm prevention assay show that bovine mastitis isolate DPC5246 is capable of forming a mature biofilm of 3.9 × 10^6^ CFU/mL in under 24 h.

Disruption of staphylococcal biofilms by phage lysins has previously been reported by Sass and Son [37, 42]. While these studies involved the complete endolysin protein with multiple domains, CHAP_ K_ is a truncated form of a natural endolysin (Lys K). It contains one lytic domain and only 33% of the original protein. Due to its lower molecular weight, CHAP_ K_ is predicted to have a lower chance of inducing a humoral immune response [46]. Also, because it is smaller CHAP_<?brm?>K<?erm?>_ is more efficiently over expressed in the recombinant *E. coli* strain compared with the full endolysin; problems of aggregation, which have been encountered with over-expression of cow udders full endolysins are greatly reduced. Lysins with just one catalytic domain, such as CHAP_ K _, may run the risk of being more susceptible to development of host resistance, than a protein with multiple catalytic domains. However, because of the position at which CHAP_ K_ cleaves the peptidoglycan (between the characteristic *S. aureus* pentaglycine bridge and the D-alanine of the tetrapeptide crossink [47]), the possibility of developing resistance seems unlikely but cannot be ruled out. In the earlier studies which reported staphylococcal biofilm disruption by phage lysins, biofilm staining was the sole method used to estimate the efficacy of the enzymatic treatment. Our study combines the staining approach with viable plate counting in order to more accurately represent the effect of the lysin on biofilm forming cells. 

At a concentration of 31.25 *μ*g mL^−1^, CHAP_ K_ completely prevented the formation of *S. aureus* biofilms. This result demonstrates the potential of applying CHAP_ K_ as a spray for decontamination of food contact surfaces or of cow udders as a preventative measure for bovine mastitisCHAP_ K _. could also be employed as a coating agent on medical implants such as catheters to prevent the adherence of staphylococci and subsequent biofilm formation and infection. Previous studies have shown that coating medical implants with antibacterial agents can be effective in preventing formation of biofilms [61–63]. Previous work by our group demonstrated that CHAP_ K_ is also effective as a biocidal agent against several pathogenic species of *Staphylococcus* including the well known biofilm former *S. epidermidis *and all known clonal types of MRSA, and thus can also be considered as a useful antimicrobial agent for prevention or treatment of infections caused by these species. A previous publication by our group on the characterisation of CHAP_ K_ demonstrated that the lysin is active over a broad range of temperatures and pH and was not seen to be susceptible to degradation by multiple freeze thawing steps [48]. The robustness of the lysin makes it attractive for commercialisation and utilisation as a decontaminating agent.

The two main reservoirs of *S. aureus *on animals are the skin and mucosal membranes. Infection can often originate from commensal flora, especially in veterinary and hospital settings, as is the case with both bovine and human mastitis [64, 65]. The present study demonstrated the potential of CHAP_ K_ as a decolonisation agent for the removal of *S. aureus *from the surface of mammalian skin. When applied as a spray, CHAP_ K_ eliminated 99% of *S. aureus* DPC5246 from skin in 30 min. The results of the experiment suggested that CHAP_ K_ could be included in bovine teat-dip solution for reduction of mastitis causing staphylococci on the udder prior to and after milking in dairy farms. In addition, treatment of human skin with CHAP_ K_ prior to surgery may help prevent serious nosocomial infections. 

In conclusion, our data demonstrates the potential of a novel but natural anti-staphylococcal agent to prevent economically important veterinary infections, nosocomial staphylococcal infections and also reduce biofilm formation in processing systems. 

## References

[1] Gruet P, Maincent P, Berthelot X, Kaltsatos V (2001). Bovine mastitis and intramammary drug delivery: review and perspectives. *Advanced Drug Delivery Reviews*.

[2] Lowy FD (1998). *Staphylococcus aureus* infections. *The New England Journal of Medicine*.

[3] Holečková B, Holoda E, Fotta M, Kalináčová V, Gondol’ J, Grolmus J (2002). Occurrence of enterotoxigenic *Staphylococcus aureus* in food. *Annals of Agriculture and Environmental Medicine*.

[4] Gutierrez D, Delgado S, Vazquez-Sanchez D (2012). Incidence of *Staphylococcus aureus* and analysis of bacterial-associated communities on food industry surfaces. *Applied and Environmental Microbiology*.

[5] Herrera JJR, Cabo ML, González A, Pazos I, Pastoriza L (2007). Adhesion and detachment kinetics of several strains of *Staphylococcus aureus* subsp. aureus under three different experimental conditions. *Food Microbiology*.

[6] Palá TR, Sevilla A (2004). Microbial contamination of carcasses, meat, and equipment from an Iberian pork cutting plant. *Journal of Food Protection*.

[7] Eady EA, Cove JH (2003). Staphylococcal resistance revisited: community-acquired methicillin resistant *Staphylococcus aureus*—an emerging problem for the management of skin and soft tissue infections. *Current Opinion in Infectious Diseases*.

[8] Lowy FD (2003). Antimicrobial resistance: the example of *Staphylococcus aureus*. *Journal of Clinical Investigation*.

[9] Donlan RM, Costerton JW (2002). Biofilms: survival mechanisms of clinically relevant microorganisms. *Clinical Microbiology Reviews*.

[10] Langsrud S, Sidhu MS, Heir E, Holck AL (2003). Bacterial disinfectant resistance—a challenge for the food industry. *International Biodeterioration and Biodegradation*.

[11] Bridier A, Briandet R, Thomas V, Dubois-Brissonnet F (2011). Resistance of bacterial biofilms to disinfectants: a review. *Biofouling*.

[12] Dunne WM (2002). Bacterial adhesion: seen any good biofilms lately?. *Clinical Microbiology Reviews*.

[13] O’Grady NP, Alexander M, Dellinger EP (2002). Guidelines for the prevention of intravascular catheter-related infections. *Infection Control and Hospital Epidemiology*.

[14] Marques SC, Rezende JDGOS, Alves LADF (2007). Formation of biofilms by *Staphylococcus aureus* on stainless steel and glass surfaces and its resistance to some selected chemical sanitizers. *Brazilian Journal of Microbiology*.

[15] Olson ME, Ceri H, Morck DW, Buret AG, Read RR (2002). Biofilm bacteria: formation and comparative susceptibility to antibiotics. *Canadian Journal of Veterinary Research*.

[16] Melchior MB, Vaarkamp H, Fink-Gremmels J (2006). Biofilms: a role in recurrent mastitis infections?. *Veterinary Journal*.

[17] Aparna MS, Yadav S (2008). Biofilms: microbes and disease. *Brazilian Journal of Infectious Diseases*.

[18] Costerton JW, Stewart PS, Greenberg EP (1999). Bacterial biofilms: a common cause of persistent infections. *Science*.

[19] Stewart PS (2002). Mechanisms of antibiotic resistance in bacterial biofilms. *International Journal of Medical Microbiology*.

[20] Nelson DC, Schmelcher M, Rodriguez-Rubio L (2012). Endolysins as antimicrobials. *Advances in Virus Research*.

[21] Fenton M, Ross P, Mcauliffe O, O’Mahony J, Coffey A (2010). Recombinant bacteriophage lysins as antibacterials. *Bioengineered Bugs*.

[22] Rodriguez-Rubio L, Martinez B, Donovan DM, Rodriguez A, García P (2012). Bacteriophage virion-associated peptidoglycan hydrolases: potential new enzybiotics. *Critical Reviews in Microbiology*.

[23] Fischetti VA (2010). Bacteriophage endolysins: a novel anti-infective to control Gram-positive pathogens. *International Journal of Medical Microbiology*.

[24] Szweda P, Schielmann M, Kotlowski R, Gorczyca G, Zalewska M, Milewski S (2012). Peptidoglycan hydrolases-potential weapons against *Staphylococcus aureus*. *Appl Microbiol Biotechnol*.

[25] Schuch R, Nelson D, Fischetti VA (2002). A bacteriolytic agent that detects and kills Bacillus anthracis. *Nature*.

[26] Loessner MJ (2005). Bacteriophage endolysins—current state of research and applications. *Current Opinion in Microbiology*.

[27] Fischetti VA (2005). Bacteriophage lytic enzymes: novel anti-infectives. *Trends in Microbiology*.

[28] Pastagia M, Euler C, Chahales P, Fuentes-Duculan J, Krueger JG, Fischetti VA (2011). A novel chimeric lysin shows superiority to mupirocin for skin decolonization of methicillin-resistant and -sensitive *Staphylococcus aureus* strains. *Antimicrobial Agents and Chemotherapy*.

[29] Kelly D, McAuliffe O, Ross RP, Coffey A (2012). Prevention of *Staphylococcus aureus* biofilm formation and reduction in established biofilm density using a combination of phage K and modified derivatives. *Letters in Applied Microbiology*.

[30] Lenski RE (1988). Dynamics of interactions between bacteria and virulent bacteriophage. *Advances in Microbial Ecology*.

[31] Coffey A, Ross RP (2002). Bacteriophage-resistance systems in dairy starter strains: molecular analysis to application. *Antonie van Leeuwenhoek*.

[32] Labrie SJ, Samson JE, Moineau S (2010). Bacteriophage resistance mechanisms. *Nature Reviews Microbiology*.

[33] Loeffler JM, Nelson D, Fischetti VA (2001). Rapid killing of Streptococcus pneumoniae with a bacteriophage cell wall hydrolase. *Science*.

[34] Fischetti VA (2003). Novel method to control pathogenic bacteria on human mucous membranes. *Annals of the New York Academy of Sciences*.

[35] O’Flaherty S, Coffey A, Meaney W, Fitzgerald GF, Ross RP (2005). The recombinant phage lysin LysK has a broad spectrum of lytic activity against clinically relevant staphylococci, including methicillin-resistant *Staphylococcus aureus*. *Journal of Bacteriology*.

[36] Yokoi KJ, Kawahigashi N, Uchida M (2005). The two-component cell lysis genes holWMY and lysWMY of the Staphylococcus warneri M phage *ϕ*WMY: cloning, sequencing, expression, and mutational analysis in *Escherichia coli*. *Gene*.

[37] Sass P, Bierbaum G (2007). Lytic activity of recombinant bacteriophage *φ*11 and *φ*12 endolysins on whole cells and biofilms of *Staphylococcus aureus*. *Applied and Environmental Microbiology*.

[38] Rashel M, Uchiyama J, Ujihara T (2007). Efficient elimination of multidrug-resistant *Staphylococcus aureus* by cloned lysin derived from bacteriophage *ϕ*MR11. *Journal of Infectious Diseases*.

[39] Obeso JM, Martínez B, Rodríguez A, García P (2008). Lytic activity of the recombinant staphylococcal bacteriophage ΦH5 endolysin active against *Staphylococcus aureus* in milk. *International Journal of Food Microbiology*.

[40] Gu J, Xu W, Lei L (2011). LysGH15, a novel bacteriophage lysin, protects a murine bacteremia model efficiently against lethal methicillin-resistant *Staphylococcus aureus* infection. *Journal of Clinical Microbiology*.

[41] Yoong P, Schuch R, Nelson D, Fischetti VA (2004). Identification of a broadly active phage lytic enzyme with lethal activity against antibiotic-resistant *Enterococcus faecalis* and *Enterococcus faecium*. *Journal of Bacteriology*.

[42] Son JS, Lee SJ, Jun SY (2010). Antibacterial and biofilm removal activity of a podoviridae *Staphylococcus aureus* bacteriophage SAP-2 and a derived recombinant cell-wall-degrading enzyme. *Applied Microbiology and Biotechnology*.

[43] Jun SY, Jung GM, Son JS, Yoon SJ, Choi YJ, Kang SH (2011). Comparison of the antibacterial properties of phage endolysins SAL-1 and LysK. *Antimicrobial Agents and Chemotherapy*.

[44] Schmelcher M, Powell AM, Becker SC, Camp MJ, Donovan DM (2012). Chimeric phage lysins act synergistically with lysostaphin to kill mastitis-causing *Staphylococcus aureus* in murine mammary glands. *Applied and Environmental Microbiology*.

[45] Idelevich EA, von Eiff C, Friedrich AW (2011). In vitro activity against *Staphylococcus aureus* of a novel antimicrobial agent, PRF-119, a recombinant chimeric bacteriophage endolysin. *Antimicrobial Agents and Chemotherapy*.

[46] Horgan M, O’Flynn G, Garry J (2009). Phage lysin LysK can be truncated to its CHAP domain and retain lytic activity against live antibiotic-resistant staphylococci. *Applied and Environmental Microbiology*.

[47] Becker SC, Dong S, Baker JR, Foster-Frey J, Pritchard DG, Donovan DM (2009). LysK CHAP endopeptidase domain is required for lysis of live staphylococcal cells. *FEMS Microbiology Letters*.

[48] Fenton M, Ross RP, McAuliffe O, O'Mahony J, Coffey A (2011). Characterization of the staphylococcal bacteriophage lysin CHAP_K_. *Journal of Applied Microbiology*.

[49] O’Flaherty S, Coffey A, Meaney WJ, Fitzgerald GF, Ross RP (2005). Inhibition of bacteriophage K proliferation on *Staphylococcus aureus* in raw bovine milk. *Letters in Applied Microbiology*.

[50] Kadurugamuwa JL, Sin L, Albert E (2003). Direct continuous method for monitoring biofilm infection in a mouse model. *Infection and Immunity*.

[51] Kadurugamuwa JL, Sin LV, Yu J (2003). Rapid direct method for monitoring antibiotics in a mouse model of bacterial biofilm infection. *Antimicrobial Agents and Chemotherapy*.

[52] Kadurugamuwa JL, Sin LV, Yu J, Francis KP, Purchio TF, Contag PR (2004). Noninvasive optical imaging method to evaluate postantibiotic effects on biofilm infection in vivo. *Antimicrobial Agents and Chemotherapy*.

[53] Fenton M, Casey PG, Hill C (2010). The truncated phage lysin CHAPk eliminates *Staphylococcus aureus* in the nares of mice. *Bioengineered Bugs*.

[54] Wu JA, Kusuma C, Mond JJ, Kokai-Kun JF (2003). Lysostaphin Disrupts *Staphylococcus aureus* and *Staphylococcus epidermidis* Biofilms on Artificial Surfaces. *Antimicrobial Agents and Chemotherapy*.

[55] Moskowitz SM, Foster JM, Emerson J, Burns JL (2004). Clinically feasible biofilm susceptibility assay for isolates of *Pseudomonas aeruginosa* from patients with cystic fibrosis. *Journal of Clinical Microbiology*.

[56] Ceri H, Olson ME, Stremick C, Read RR, Morck D, Buret A (1999). The Calgary Biofilm Device: new technology for rapid determination of antibiotic susceptibilities of bacterial biofilms. *Journal of Clinical Microbiology*.

[57] Hoopes JT, Stark CJ, Kim HA, Sussman DJ, Donovan DM, Nelson DC (2009). Use of a bacteriophage lysin, PlyC, as an enzyme disinfectant against *Streptococcus equi*. *Applied and Environmental Microbiology*.

[58] Kumar CG, Anand SK (1998). Significance of microbial biofilms in food industry: a review. *International Journal of Food Microbiology*.

[59] Hell É, Giske CG, Nelson A, Römling U, Marchini G (2010). Human cathelicidin peptide LL37 inhibits both attachment capability and biofilm formation of *Staphylococcus epidermidis*. *Letters in Applied Microbiology*.

[60] Furukawa S, Akiyoshi Y, Komoriya M, Ogihara H, Morinaga Y (2010). Removing *Staphylococcus aureus* and *Escherichia coli* biofilms on stainless steel by cleaning-in-place (CIP) cleaning agents. *Food Control*.

[61] Bruellhoff K, Fiedler J, Moller M, Groll J, Brenner RE (2010). Surface coating strategies to prevent biofilm formation on implant surfaces. *The International Journal of Artificial Organs*.

[62] van Heerden J, Turner M, Hoffmann D, Moolman J (2009). Antimicrobial coating agents: can biofilm formation on a breast implant be prevented?. *Journal of Plastic, Reconstructive and Aesthetic Surgery*.

[63] Bernthal NM, Stavrakis AI, Billi F (2010). A mouse model of post-arthroplasty *Staphylococcus aureus* joint infection to evaluate in vivo the efficacy of antimicrobial implant coatings. *PloS ONE*.

[64] Gong JQ, Lin L, Lin T (2006). Skin colonization by *Staphylococcus aureus* in patients with eczema and atopic dermatitis and relevant combined topical therapy: a double-blind multicentre randomized controlled trial. *British Journal of Dermatology*.

[65] Kluytmans J, van Belkum A, Verbrugh H (1997). Nasal carriage of *Staphylococcus aureus*: epidemiology, underlying mechanisms, and associated risks. *Clinical Microbiology Reviews*.

